# Synthesis and thermoelectric properties of Rashba semiconductor BiTeBr with intensive texture

**DOI:** 10.1007/s12598-018-1027-9

**Published:** 2018-04-07

**Authors:** Jia-Zhan Xin, Chen-Guang Fu, Wu-Jun Shi, Guo-Wei Li, Gudrun Auffermann, Yan-Peng Qi, Tie-Jun Zhu, Xin-Bing Zhao, Claudia Felser

**Affiliations:** 1 Max Planck Institute for Chemical Physics of Solids, 01187 Dresden, Germany; 2 State Key Laboratory of Silicon Materials, School of Materials Science and Engineering, Zhejiang University, Hangzhou, 310027 China; 3 School of Physical Science and Technology, ShanghaiTech University, Shanghai, 200031 China

**Keywords:** Bismuth tellurohalides, BiTeBr, Thermoelectric properties, Texture

## Abstract

Bismuth tellurohalides with Rashba-type spin splitting exhibit unique Fermi surface topology and are developed as promising thermoelectric materials. However, BiTeBr, which belongs to this class of materials, is rarely investigated in terms of the thermoelectric transport properties. In the study, polycrystalline bulk BiTeBr with intensive texture was synthesized via spark plasma sintering (SPS). Additionally, its thermoelectric properties above room temperature were investigated along both the in-plane and out-plane directions, and they exhibit strong anisotropy. Low sound velocity along two directions is found and contributes to its low lattice thermal conductivity. Polycrystalline BiTeBr exhibits relatively good thermoelectric performance along the in-plane direction, with a maximum dimensionless figure of merit (ZT) of 0.35 at 560 K. Further enhancements of ZT are expected by utilizing systematic optimization strategies.

## Introduction

Solid-state thermoelectric (TE) materials enable the direct conversion of waste heat into electric power and provide a possible solution for increased energy demands [1–6]. The efficiency of a TE material is generally gauged by its dimensionless figure of merit, ZT = *α*^2^*σT*/(*κ*_e_ + *κ*_L_), where *α* denotes the Seebeck coefficient, *σ* denotes electrical conductivity, *κ*_e_ and *κ*_L_ denote the electronic and lattice contributions to the total thermal conductivity (*κ*), respectively, and *T* denotes the absolute temperature [2]. Extant studies aim to obtain higher ZT and mainly focus on two aspects. The first aspect involves engineering the electronic structure to enhance the power factor (PF = *α*^2^*σ*) [7, 8], i.e., by increasing band degeneracy [9, 10], inducing resonant levels [11] and reducing band effective mass [12, 13]. The other aspect involves obtaining lower *κ*_L_ [14] by introducing multiscale phonon scattering centers [15–17] or pursuing new materials with intrinsically low *κ*_L_ [18–20].

Layered ternary bismuth tellurohalides (BiTeX with X = I, Br, Cl) with giant Rashba-type spin splitting have recently received widespread interest as future spintronic applications and have been explored as topological superconductors [21, 22]. Given the spin–orbital coupling and inversion asymmetry, BiTeX exhibits unique Fermi surface topology, reduced dimensionality for the electronic density of state, and thus unusual relativistic physical properties [23, 24]. A recent study indicated that unique Fermi surface and complex non-parabolic band structures are favorable for high TE performance [25]. Thus, semiconductor BiTeX with Rashba-type spin splitting displays promising TE transport properties.

With respect to the BiTeX system, BiTeI attracted maximum attention in terms of the TE transport investigation, and this is partly due to the strongest Rashba-type spin splitting. Wu et al. [26] found a two-dimensional thermopower in BiTeI due to the spin-splitting-induced constant density of states, and this exceeds that in spin-degenerate bands. With increase in atomic number of the halogen element, the lattice thermal conductivity of BiTeX decreases due to the higher average atomic mass (*κ*_L_ is approximately 1 W·m^−1^·K^−1^ at RT) [27, 28]. Nevertheless, with respect to the aforementioned three types, BiTeI is significantly affected by intrinsic point defects [29]. The electron concentration of single-crystalline BiTeI (4.6 × 10^19^ cm^−3^) significantly exceeds the optimized value (approximately 5 × 10^18^ cm^−3^) [27] as estimated from the single parabolic band model [8, 30]. Additionally, additional defect scattering leads to relatively small carrier mobility, and thereby a low power factor (approximately 5 μW·cm^−1^·K^−2^) [27, 29]. Wu et al. [29] indicated that Cu-intercalation in BiTeI substantially alters the equilibria of defect reactions and leads to increases in carrier mobility and consequently an enhanced power factor. Furthermore, the alloying of Br broadens the band gap of BiTeI and this leads to diminished thermally activated minority carriers and improved ZT at high temperatures [31]. In a single-crystalline form, BiTeCl was grown by using a topotactic method and its TE transport properties below room temperature were investigated. A maximum power factor corresponding to 18 μW·cm^−1^·K^−2^ and ZT of 0.17 was reported [28]. However, the TE properties of BiTeCl were observed as deteriorating with respect to time, which indicates that the system is not stable [28]. The TE properties of BiTeI and BiTeBr single crystals were investigated below room temperature in which BiTeBr exhibits a value of ZT that is almost twice that of BiTeI [27].

Thus, BiTeX systems are still not fully examined for their TE properties, especially in polycrystalline form and above room temperature. Additionally, the inter-layer interaction for BiTeX is due to the van der Waals force. Thus, strong anisotropy should exist for their transport properties, and this requires further investigation [29]. When compared to BiTeI, BiTeBr exhibits a higher band gap, and this acts to suppress the thermal activation of minority carrier. In the study, polycrystalline BiTeBr with intensive texture was synthesized by spark plasma sintering (SPS). The anisotropic TE properties along in-plane and out-plane directions were investigated. The results indicate that the in-plane direction of polycrystalline BiTeBr exhibits relatively good thermoelectric performance with a maximum dimensionless figure of merit ZT of approximately 0.35 at 560 K.

## Experimental and theoretical methods

In the first set of experiments, BiTeBr_1−*x*_Cl_*x*_ (0 ≤ *x* ≤ 1) were synthesized and their TE transport properties were examined. The results indicate that the specimens with high Cl content are sensitive to moisture and cannot be kept in air for a long period. A similar phenomenon was also reported in a previous study on BiTeCl single crystal [28]. Therefore, in the following study, we only focused on investigating the TE transport properties of more stable BiTeBr and BiTeBr_0.75_Cl_0.25_. In the typical synthesis of polycrystalline specimens with nominal composition BiTeBr and BiTeBr_0.75_Cl_0.25_, stoichiometric amounts of elemental Bi (piece, 99.999%), Te (piece, 99.99%), BiBr_3_ (powder, 99.9%) and BiCl_3_ (powder, 99.8%) were weighed and loaded in quartz tubes in a glove box. The quartz tubes were sealed under partial Ar pressure and then placed into a furnace. The quartz tubes were first heated to 600 °C, kept for 10 h, then cooled down to 400 °C and kept for 7 days. The obtained ingots were manually crushed and then placed into graphite dies with an inner diameter of 8 mm. The dies were placed into a SPS instrument (Fuji, Japan) and compacted at 340 °C for 4 min under 80 MPa in vacuum. Finally, bulk samples with a diameter of 8 mm and a thickness of approximately 11 mm were obtained.

Powder X-ray diffraction (XRD) measurement was performed with Cu Kα radiation at room temperature to identify the phase purity and crystal structure by using an image-plate Huber G670 Guinier camera with a diffraction range of 10° ≤ 2*θ* ≤ 100° with a step of 0.005°. The microstructure of the samples was examined by using scanning electron microscope (SEM, FEI Quanta 200 F). Differential thermal analysis (DTA) and thermal gravimetric analysis (TG) were performed (DTA/TG, NETZSCH STA 449F3) to identify the thermal stability. The transport properties of the samples were measured both along the in-plane (*ab* plane) and out-plane (*c* axis) directions. The Seebeck coefficient and resistivity were measured by using an ULVAC ZEM-3 system. The thermal diffusivity was determined by using laser flash analysis (LFA 457, Netzsch). The thermal conductivity was calculated by using the equation *κ* = *DρC*_p_, where *D* denotes the thermal diffusivity, *ρ* denotes the density and *C*_p_ denotes the specific heat that is estimated by using the Dulong–Petit value. The estimated measurement uncertainties are 3% for electrical conductivity, 7% for the Seebeck coefficient and 3% for thermal diffusivity. The Hall coefficients (*R*_H_) at room temperature were determined from the slope of the Hall resistivity as a function of magnetic field measured by using the physical property measurement system (PPMS, Quantum Design). The carrier concentration (*n*_H_) was calculated by using *n*_H_ = 1/*eR*_H_ (estimated error within ± 10%), where *e* denotes the unit charge. The carrier mobility (*μ*_H_) was calculated by using *μ*_H_ = *σR*_H_. Normal and shear ultrasonic measurements were performed at room temperature by using input from a Panametrics 5052 pulser/receiver with a filter at 0.03 MHz. The response was recorded via a Tektronix TDS5054B-NV digital oscilloscope. The high-resolution mode was employed for the longitudinal speed of sound (*v*_L_), and an averaging mode (16 wave forms) was utilized for the transverse speed-of-sound (*v*_T_) measurements. The Debye temperature (*θ*_D_) was calculated by $\theta_{\text{D}} \, = \,\hbar v_{\text{s}} \left( {6\uppi^{2} n} \right)^{1/3} /k_{\text{B}}$, where $\hbar$ denotes the reduced Planck constant, *k*_B_ denotes the Boltzmann constant, *n* denotes the atom number in a unit volume, and *v*_s_ denotes the average speed of sound calculated from *v*_s_ = (*v*_L_^−3^/3 + 2*v*_T_^−3^/3)^−1/3^. The measured sound velocity and the Debye temperature for all the specimens are shown in Table 1.

**Table 1:** Lattice parameter, orientation factor, Hall carrier concentration, Hall mobility (at 300 K), sound velocity and Debye temperature for sintered BiTeBr and BiTeBr_0.75_Cl_0.25_

Composition	Lattice parameter/nm		Orientation factor (*F*)	Hall carrier concentration (*n*_H_)/10^19^ cm^−3^	Hall mobility, *ab* (*µ*_H_)/(cm^2^·V^−1^·s^−1^)	Sound velocity*, ab*/(m·s^−1^)		Sound velocity*, c*/(m·s^−1^)		Debye temperature/K	
	*a*	*c*				*v*_L_	*v*_T_	*v*_L_	*v*_T_	*θ*_D, *ab*_	*θ*_D, *c*_
BiTeBr	0.4251	0.6449	0.362	1.44	244	2633	1681	2138	1378	170	139
BiTeBr_0.75_Cl_0.25_	0.4255	0.6441	0.376	1.52	139	2333	1333	2308	1111	137	115

Density functional theory calculations (DFT) were performed by using the Vienna Ab-initio Simulation Package (VASP) [32] to investigate the electronic properties. The interactions between the valence electrons and ion cores were described by using the projector-augmented wave method [33, 34]. The exchange and correlation energy were formulated by using the generalized gradient approximation (GGA) with the Perdew–Burke–Ernzerhof scheme [35]. The plane-wave basis cutoff energy was set as 216 eV by default. The Γ-centered *k* points with 0.3 nm^−1^ spacing were used for the first Brillouin-zone sampling. The spin-orbit coupling (SOC) was included in the calculation.

## Results and discussion

### Microstructure and thermal stability

**Fig. 1:**
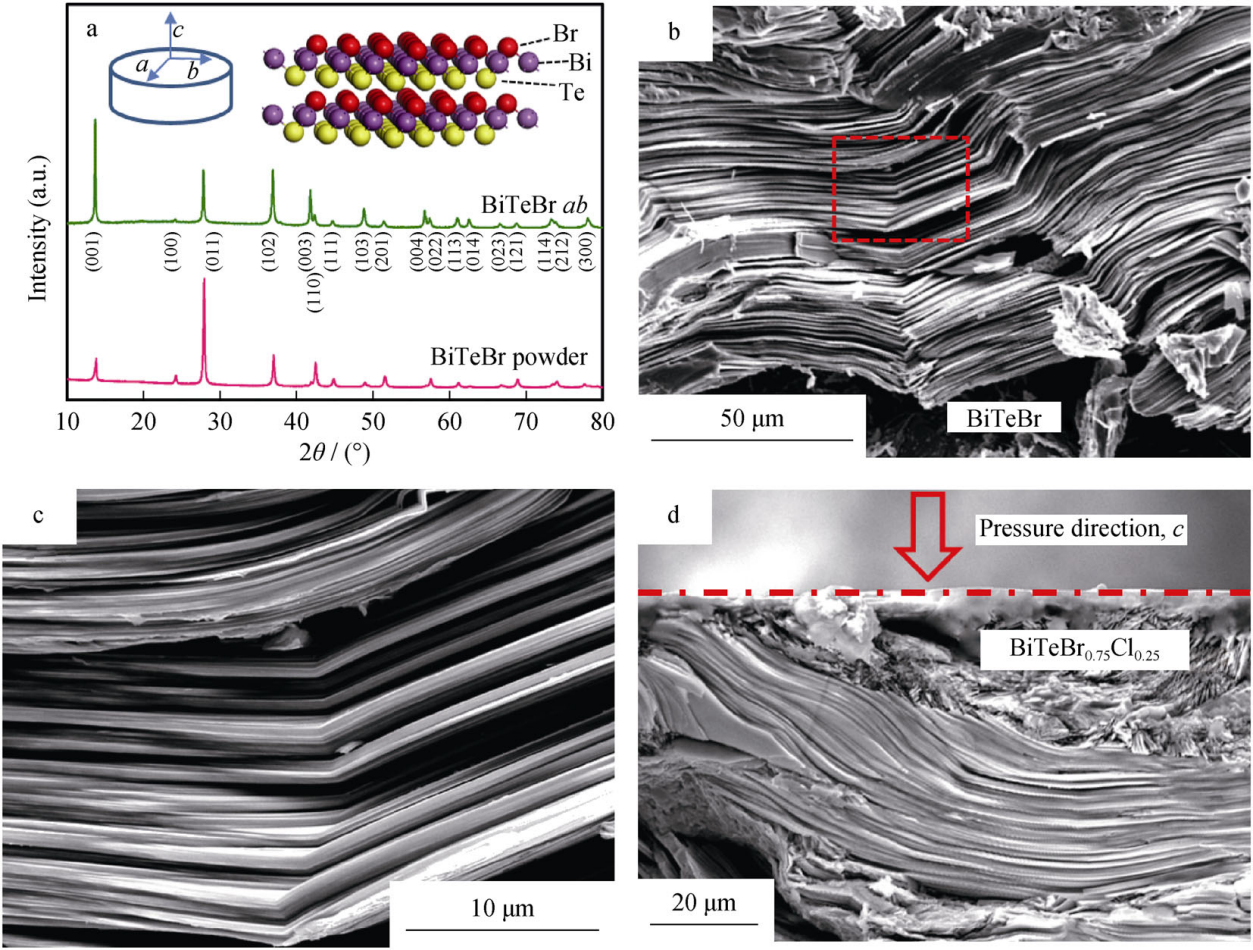
**a** Powder XRD and bulk XRD patterns for BiTeBr (inset on left displaying marks for different directions for bulk specimens, and inset on right being a schematic for crystal structure of BiTeBr, **b** SEM fractographs of cross sections parallel to pressing direction for BiTeBr, **c** enlarged view of zone in red dotted box in **b** and **d** SEM image for BiTe_0.75_Cl_0.25_ with a free surface perpendicular to SPS pressure

XRD patterns of powder and bulk BiTeBr are shown in Fig. 1a. All major peaks are indexed to the 2H-CdI_2_ type structure (space group No. 156, *P*3*m*1). The lattice parameter of BiTeBr is estimated as *a* = 0.4251 nm, *c* = 0.6449 nm, and this is consistent with those obtained in the previous experimental studies [36]. In the following section, we clarify this and define *ab* as referring to the specimens in which the in-plane direction is perpendicular to the SPS pressure direction, while the direction parallel to the SPS pressure is denoted as *c*. XRD pattern of the specimen in the bulk form is also shown in Fig. 1a and indicates an intensive texture. XRD intensity of (00*l*) peaks for the bulk specimen is several times higher than that of the powder XRD result. In order to quantitatively address the texture degree, the orientation factor (*F*) of (00*l*) planes is calculated based on the Lotgering method [37], as shown in Table 1. A significantly high *F* corresponding to 0.36 is obtained for BiTeBr, and this is comparable to that of hot-deformed Bi_2_Te_3_ [38, 39]. This type of an intensive texture is also observed in the SEM sectional views as shown in Fig. 1b, d. The layered topography of the sintered material is evident although it is twisted. Thin sheets with thickness corresponding to hundreds of nanometers are distinguished, as shown in Fig. 1c.

**Fig. 2:**
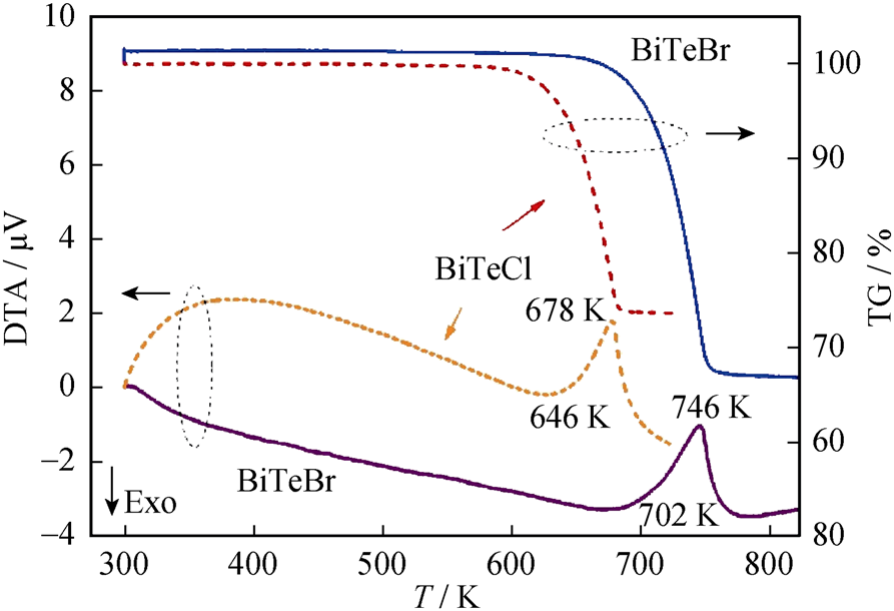
DTA/TG analysis of BiTeBr and BiTeCl specimens

DTA and TG were performed to confirm the thermal stability of BiTeBr and BiTeCl and their working temperature range as TE materials. Similar curves are observed for both materials, as shown in Fig. 2. With respect to the BiTeCl specimen, the endothermic peak at 678 K corresponds to the decomposition of BiTeCl into Bi_2_Te_3_ and BiCl_3_, and this is consistent with the result obtained in previous study [40]. As shown in TG curve, the BiTeCl specimen begins to decompose at approximately 600 K. Thus, the TE transport properties in the study were only measured up to 600 K. DTA result for BiTeBr exhibits a decomposition reaction at 746 K, while the previous result only indicates a melting point at 800 K during the heating process without any decomposition of BiTeBr prior to that [41].

### Calculated band structure

**Fig. 3:**
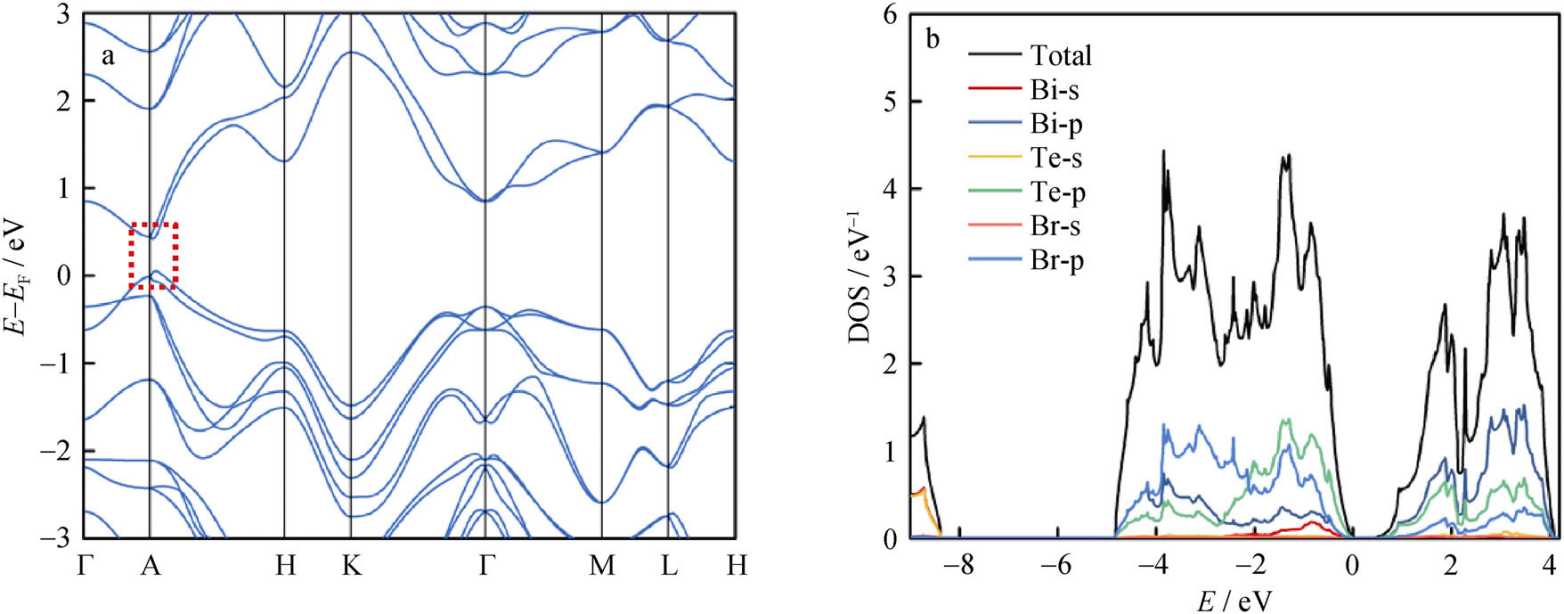
**a** Calculated band structure (Rashba spin splitting band feature observed in band edge, marked in red dotted box) and **b** total DOS as well as partial DOS for BiTeBr system

The DFT calculated band structure for BiTeBr is shown in Fig. 3a. The energy band gap (*E*_g_) is estimated as approximately 0.37 eV, and this is consistent with the value obtained from optical measurements [31]. The Rashba-type spin splitting is found near the band edge as indicated by the red dotted box. The Rashba energy (a quantitative description for the extent of Rashba-type spin splitting) for BiTeBr is approximately 0.03 eV, and this is lower than that of BiTeI (approximately 0.1 eV) [42]. Figure 3b shows the total density of state (DOS) of BiTeBr, and the partial DOS for all the related orbitals. As shown from the results, the valence band is dominated by the p-orbit of Te atom, while both the p-orbits of Bi and Te significantly contribute to the conduction band.

### Electrical properties

**Fig. 4:**
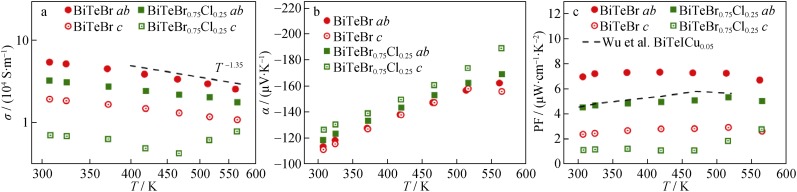
Temperature dependencies of **a** electrical conductivity (*σ*), **b** Seebeck coefficient (*α*) and **c** power factor (PF) for BiTeBr and BiTeBr_0.75_Cl_0.25_ specimens along *ab* and *c* directions in conjunction with experimental data from Wu et al. [29]

The temperature dependencies of electrical conductivity and Seebeck coefficient for the specimens are shown in Fig. 4. Typical degenerated semiconductor transport behavior is observed for all the specimens in which *σ* decreases, while the absolute value of *α* increases with the increase in the temperature (with the exception of the BiTeBr_0.75_Cl_0.25_ along the *c* direction above 470 K). A negative value of *α* indicates n-type conduction with electrons as the major carrier. Additionally, the emergence of obvious bipolar conduction in these specimens is absent as indicated by the temperature dependence of *α*. The *σ* decreases with the increase in temperature and follows a *T*^−1.35^ relationship, indicating that acoustic phonon scattering dominates the electron transport [43].

The electrical conductivity along the *ab* direction is more than twice that in the *c* direction and indicates strong anisotropy for the sintered specimens. The Seebeck coefficient for these two directions differs slightly and is similar to the case for Bi_2_Te_3_ [44]. Additionally, the alloying of Cl in BiTeBr reduces the electrical conductivity. This is due to the enhanced alloying scattering. As seen in Table 1, the Hall electron mobility of BiTeBr_0.75_Cl_0.25_ is only half of the value of BiTeBr though their electron concentrations are very close.

The power factor of all the specimens is shown in Fig. 4c. The BiTeBr specimen exhibits a maximum PF (approximately 8 μW·cm^−1^·K^−2^) along *ab* direction, and this exceeds that of state-of-art Cu-intercalated BiTeI [29]. This indicates that BiTeBr exhibits better electrical properties compared with BiTeI, although the Rashba energy of the former is lower [42]. This is only corresponding to the result for the pristine BiTeBr, and thus further enhancements in its power factor might be realized through carrier optimization or band engineering.

### Thermal conductivity and ZT

**Fig. 5:**
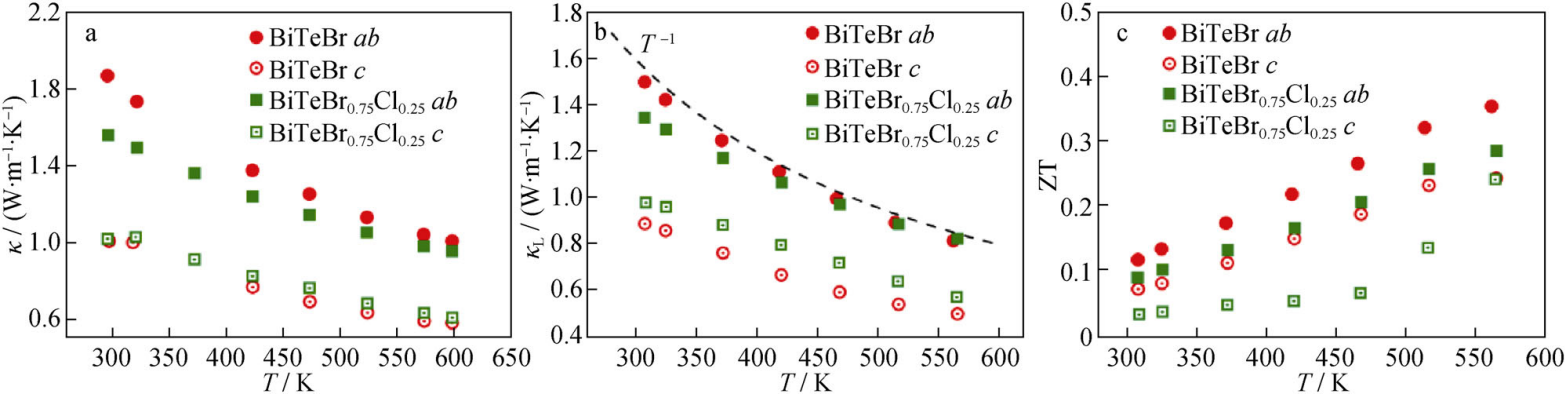
Temperature dependences of **a** thermal conductivity (*κ*), **b** lattice thermal conductivity (*κ*_L_) and **c** dimensionless figure of merit (ZT) for the BiTeBr and BiTeBr_0.75_Cl_0.25_ specimens along *ab* and *c* directions

The temperature dependencies of thermal conductivity for all the specimens are shown in Fig. 5a. Strong anisotropy is also found. The thermal conductivity along *c* direction is 50% lower than that along *ab* direction. The lattice thermal conductivity was calculated by deducing the electrical contribution, *κ*_e_ = *LσT*, in which the Lorenz number (*L*) is estimated based on the single parabolic band model when acoustic phonon scattering dominates [43, 45]. The magnitude of the calculated *L* is approximately 1.8 × 10^−8^ V^2^·K^−2^. The lattice thermal conductivity follows a *T*^−1^ trend with increase in the temperature as shown in Fig. 5b, thereby, indicating that the three phonon Umklapp process dominates the phonon transport [15]. A low *κ*_L_ corresponding to 0.8 W·m^−1^·K^−1^ at 560 K is obtained for BiTeBr specimen along *ab* direction. However, the reduction in lattice thermal conductivity along *ab* direction caused by Cl substitution is limited (10% reduction of *κ*_L_ at room temperature) and even negligible at elevated temperatures. With respect to *c* direction, BiTeBr displays a significantly lower *κ*_L_ compared to that along *ab* plane, and this is similar to other layered TE materials [46, 47]. The overall low *κ*_L_ in both the *ab* and *c* directions of BiTeBr is considered as partly related to its intrinsic low sound velocity, and this indicates weak chemical bonding in the system [20, 26]. The average sound velocity (*v*_s_) of BiTeBr along the *ab* direction is 1850 m·s^−1^, and this is comparable to those of the other potentially promising TE materials with intrinsically low *κ*_L_ (e.g., *v*_s_ of MgAgSb ≈ 1921 m·s^−1^ and *v*_s_ of Bi_2_Te_3_ ≈ 2147 m·s^−1^) [20, 48]. The Debye temperature of BiTeBr is calculated as approximately 170 K.

The dependence of ZT as a function of temperature is shown in Fig. 5c. The ZT along *ab* direction is evidently exceeds that along *c* direction due to the higher electrical conductivity. The maximum figure of merit (ZT) of 0.35 is obtained for BiTeBr specimen at 560 K. It is possible to reach a higher peak ZT if the measurement temperature is increased further. However, given the possible thermal instability, this does not extend to higher temperatures. Although Cl alloying in BiTeBr lowers the thermal conductivity, it results in a deterioration in the electrical properties and does not contribute to improving the TE performance. To further enhance ZT of BiTeBr, reducing the carrier concentration to further enhance the power factor is a promising way. Furthermore, isoelectronic alloying by using Sb could be another effective way to suppress the lattice thermal conductivity and thus improve the TE performance.

## Conclusion

In summary, polycrystalline BiTeBr-based bulk materials with intensive texture were successfully synthesized by using SPS and their thermoelectric properties above temperature were reported. Intensive texture results in anisotropic electrical and thermal transport properties. Overall, the ZT value along the in-plane direction exceeds that along the out-plane direction due to higher electrical conductivity. Low sound velocity along two directions is found in polycrystalline BiTeBr and contributes to its low lattice thermal conductivity. A maximum figure of merit (ZT) of 0.35 is obtained for BiTeBr specimen at 560 K. An increase in TE performance is expected for the new material system by further optimizing the transport properties.
